# Proof of concept for a syndromic surveillance system based on routine ambulance records in the South West of England, for the influenza season 2016/2017

**DOI:** 10.29045/14784726.2019.09.4.2.22

**Published:** 2019-09-01

**Authors:** Thilo Reich, Marcin Budka

**Affiliations:** Bournemouth University: ORCID iD: https://orcid.org/0000-0001-7705-0987; Bournemouth University: ORCID iD: http://orcid.org/0000-0003-0158-6309

**Keywords:** biosurveillance, electronic patient records, outbreak detection, pre-hospital data

## Abstract

**Introduction:**

The introduction of electronic patient records in the ambulance service provides new opportunities to monitor the population. Approximately 36% of patients presenting to English ambulance services are discharged at scene. Ambulance records are therefore an ideal data source for syndromic early event detection systems to monitor infectious disease in the pre-hospital population. It has been previously found that tympanic temperature records can be used to detect influenza outbreaks in emergency departments. This study aimed to determine whether routine tympanic temperature readings collected by ambulance crews can be used to detect seasonal influenza.

**Methods:**

Here we show that temperature readings do allow the detection of seasonal influenza before methods applied to conventional data sources. The counts of pyretic patients were used to calculate a sliding case ratio as a measurement to detect seasonal influenza outbreaks. This method does not rely on conventional thresholds and can be adapted to the data.

**Results:**

The data collected correlated with seasonal influenza. The 2016/2017 outbreak was detected up to nine weeks before other surveillance programmes. The results show that ambulance records can be a useful data source for biosurveillance systems.

**Conclusion:**

Temperature readings from routinely collected ambulance patient records can be used as a surveillance tool for febrile diseases.

## Introduction

The digitisation of ambulance healthcare records has created a large pre-hospital data source that to date is mostly untapped. South Western Ambulance Service NHS Foundation Trust (SWASFT) introduced electronic patient care records (ePCRs) in March 2015, making it possible to access and monitor all data recorded in near real-time.

Infections usually result in pyrexia (Bartfai & Conti, 2011; Hasday, Fairchild, & Shanholtz, 2000), making body temperature-based surveillance systems non-specific, but sensitive to virtually any pyrexia-causing disease. Temperature screening has been applied during outbreaks of infectious diseases, such as severe acute respiratory syndrome (SARS) (Samaan, Patel, Spencer, & Roberts, 2004; Syed, Sopwith, Regan, & Bellis, 2003). It has also been demonstrated that the monitoring of body temperature on its own makes it possible to detect outbreaks of seasonal flu in emergency departments (Bordonaro et al., 2016). However, if syndromic surveillance systems were put in place in ambulance services, it might be possible to detect outbreaks of disease while it is still in the community, prior to detection by hospital-based systems (Barishansky, 2009).

This study aimed to demonstrate that it is possible to utilise ambulance service ePCRs to detect disease outbreaks, solely based on tympanic temperature readings. The objectives of this study were:

- To establish if the pre-hospital tympanic temperature readings mirrored the seasonal influenza peak during the 2016/2017 season.
- To evaluate a method adapted from Singh, Savill, Ferguson, Robertson and Woolhouse (2010) using case ratios (CRs) and its applicability as an early event detection (EED) system when applied to pre-hospital tympanic temperature readings.

## Methods

### Data extraction

All ePCRs created between 1 January 2015 and 30 April 2017, with an incident postcode matching the county of Devon or Cornwall, were eligible for inclusion. Records without valid postcodes were excluded. The postcode, record creation date, tympanic temperature and age were requested and provided by the SWASFT Clinical Information and Records Office.

### Temperature measurement in South Western Ambulance Service NHS Foundation Trust

The most commonly used temperature probes within SWASFT are the Braun ThermoScan 7 IRT6520 and ThermoScan 5 IRT4520. Both devices have a measurement range of 34.0–42.2°C with an accuracy of 0.2°C between 35.5 and 42.0°C, and 0.3°C outside this range (Braun, 2007, 2013). The definition of pyrexia used in this evaluation was 37.8–42.0°C (Bordonaro et al., 2016; Obermeyer, Samra, & Mullainathan, 2017). All temperature readings outside of the manufacturer’s specifications were excluded as erroneous values.

### Data processing

All data were processed using MATLAB R2017a (The MathWorks, MA, USA). Daily and weekly counts of call volumes and pyretic patient numbers were used as a basis for all following data analyses. Due to the staggered deployment of the electronic devices within SWASFT, ePCRs were not available in all areas until 5 January 2016, so the evaluation was limited to the period between 5 January 2016 and 30 April 2017. Since the start of the 2015/2016 flu season was not captured, all detections were run against the 2016/2017 flu peak.

### Data smoothing

As the daily patient count varies considerably from one day to another resulting in a noisy time series, the data were smoothed using an exponential moving average (EMA), with an averaging window of 21 days. This window size was chosen because the incubation period of influenza can be up to 3.6 days (Lessler et al., 2009), followed by an onset of symptoms and transmission period of the virus which can last up to 10 days in hospital (Fielding, Kelly, Mercer, & Glass, 2014; Ip et al., 2015; Suess et al., 2012). This means a patient could be contagious for up to 14 days following infection. Accounting for the incubation period, a secondary patient could show symptoms 18 days after the infection of the index case. Therefore, the averaging window size was chosen to be 21 days as this allows for some leeway.

An EMA method was also used because it gives data points a greater weighting if they are closer to the present compared to samples from the more distant past (Fricker, 2010). This places greater emphasis on data from new patients rather than on older data points.

The weekly summed data were used without smoothing as well as an EMA of three weeks equivalent to three sample points.

### Baseline calculation

The weekly sums and the smoothed daily and weekly counts of pyretic patients were binned with bin-counts calculated using the Freedman–Diaconis Rule (Freedman & Diaconis, 1981). The centre of the most frequent bin range was determined and will be referred to as baseline.

### Normalisation

Figures showing variables of different scales were normalised using min–max normalisation to represent the values on a scale between 0 and 1, where the maximum value is assigned to 1.

### Reference datasets

To establish whether the seasonal influenza outbreak is detectable in the ePCR data, these data were compared to a reference dataset of weekly influenza cases in England obtained from the European Centre for Disease Prevention and Control (ECDC).

### Calculation of the modified case ratio CR_d_

The ability of an infectious agent to spread within a population can be described using the basic reproduction number or R_0_. This value indicates the mean secondary infections caused by each infected host in a naïve population without immunity against the infectious agent. R_0_ is calculated retrospectively using information about the number of contacts of each infected individual and the resulting secondary infections (Breban, Vardavas, & Blower, 2007).

Methods exist to estimate R_0_ from the progress of a disease outbreak, which rely on knowledge about the transmission characteristics of the infectious agent gathered from previous outbreaks (Althaus, 2014; Griffin, Garske, Ghani, & Clarke, 2011; Potapov, Merrill, Pybus, & Lewis, 2015). As this evaluation only focuses on abnormal temperature readings, the infection that could be responsible is not possible to determine and so cannot be compared directly to previous outbreaks. However, Singh et al. (2010) demonstrated that weekly CRs can be used as an indirect measure of R_0_ and allow detection of pandemic influenza outbreak, and so this method was adapted in this study by using several different time frames compared solely to the weekly CR.

To distinguish between different time frames used to calculate the modified CR in this evaluation, it is referred to as CR_d_ where d represents the chosen time step between observations in days, using the calculation shown in the following equation:

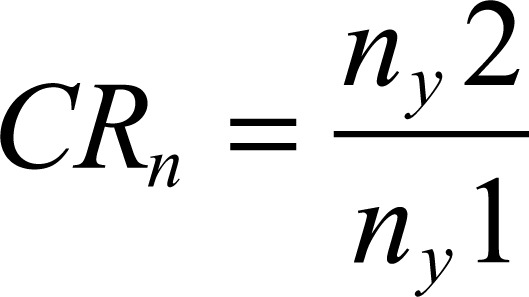



where n_y_ represents the number of pyretic patients at the days of observation with the previously defined time step between observations in days. Thus, n_y_1 represents the first observation and n_y_2 the latest. Here this method is applied to pyrexia cases as an unspecific substitute for infection.

### Outbreak definition

In this study, the outbreak definition is focused on the ascending slope, representing an increase in pyrexia case numbers. Therefore, the definition of an outbreak is the persistent transition from CR_d_ < 1 to CR_d_ > 1 indicating an increase in cases, which remains > 1 for at least one week. It has to be noted that outbreak here refers to an increase of pyrexia cases caused by an unspecified infectious agent and could be caused by several agents circulating at the same time.

A delay of one week was chosen because it includes the incubation time, meaning that secondary patients exposed to influenza should have developed pyrexia within one week (Lessler et al., 2009).

Using CR_d_ as an indication of an outbreak start is based on the assumption that a disease becomes uncontrolled once CR becomes larger than 1 (Woolhouse et al., 2001). The final change of CR_d_ to > 1, that is not followed by a recovery to < 1 within one week until the curve reaches its peak, is considered the ascending period of the outbreak.

## Results

### Data characteristics

**Figure 1:**
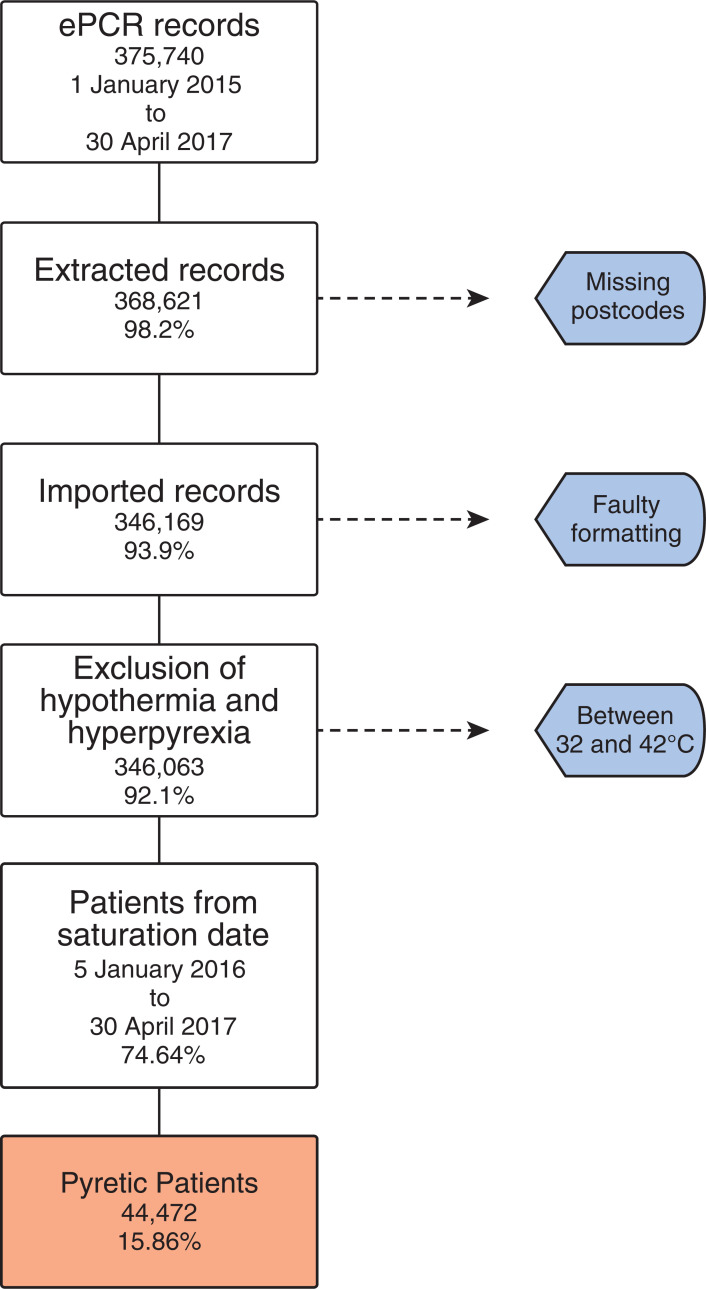
Figure 1. Summary of electronic patient care records available for analysis, including reasons for exclusion.

Note: ePCR = electronic patient care record.

Between 1 January 2015 and 30 April 2017, there were 375,740 ePCRs generated by SWASFT. Once records with missing postcodes, faulty formatting and abnormally high or low temperature values were excluded, 346,063 remained. As previously mentioned, the saturation data for ePCR deployment were not reached until 5 January 2016 and so records before this were omitted, resulting in 280,452 records available which were used for all following analyses. Of those, 44,472 met the study definition for pyrexia (Figure 1).

Temperatures recorded were in the range of 21.0–47.0°C, with a mean temperature of 36.9°C. Temperatures outside of this range were assumed to be errors. The temperature-based exclusion removed eight patients (0.0023%) with temperatures of > 42.0°C, three of those within the 42.0–42.2°C range. The lower temperature cut-off removed 98 patients (0.028%) with temperatures of < 32.0°C.

The dataset from the date of saturation was made up of 54.0% female and 46.0% male patients. The age range was 0–115 years (one outlier of 864 years was excluded), with a mean age of 60 and a median age of 68.

**Figure 2:**
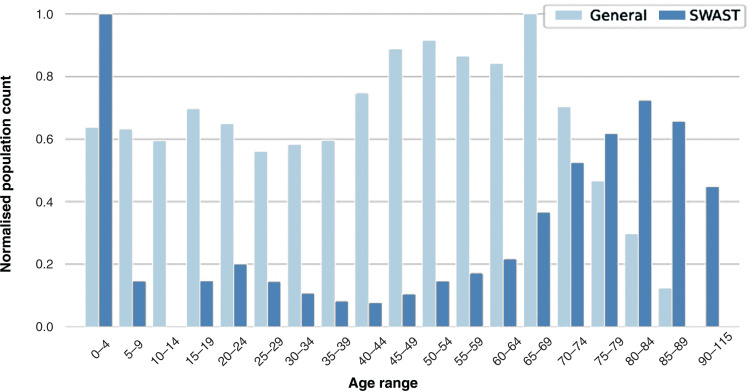
Figure 2. Age distribution for the general population in Devon and Cornwall in comparison to the patients attended by ambulance crews.

Note: SWASFT = South Western Ambulance Service NHS Foundation Trust.

The estimated population in 2016 was 779,834 for Devon and 553,687 for Cornwall. The combined population was 1,333,521 (Office for National Statistics, 2017). In comparison to the estimated age distribution of Devon and Cornwall, the SWASFT data were skewed towards the elderly and young children (Figure 2).

### Influenza detection

To establish whether seasonal influenza was detectable, weekly case numbers were compared with weekly sentinel influenza cases recorded by the ECDC in England. Sentinel surveillance data are based on a network of selected healthcare facilities, which select patients with symptoms suggesting influenza for laboratory confirmation. The non-sentinel surveillance is a passive system, using patient samples for laboratory confirmation of a variety of sources which are not necessarily from patients showing symptoms of an influenza infection (European Centre for Disease Prevention and Control, 2018).

Public Health England (PHE) monitors influenza cases with different surveillance programmes. The data are based on diagnoses from hospitals as well as from GPs. They are separated into influenza-like illnesses (ILI) and acute respiratory infections (ARI) (Public Health England, 2017).

**Figure 3:**
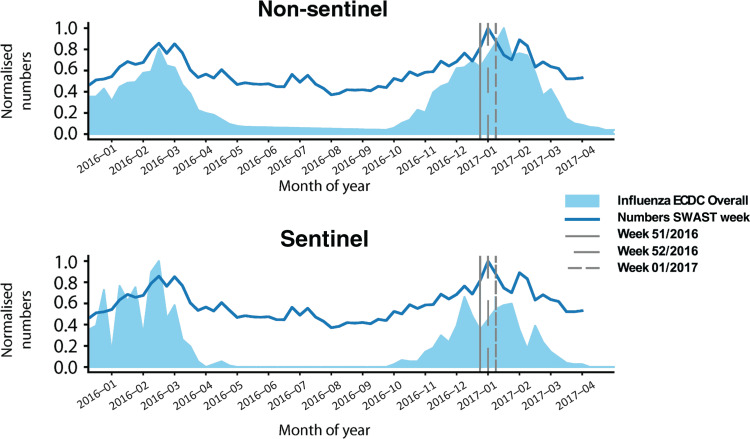
Figure 3. Normalised weekly pyrexia recorded in electronic patient care records compared to flu cases in England as reported by the European Centre for Disease Prevention and Control.

Note: ECDC = European Centre for Disease Prevention and Control; SWASFT = South Western Ambulance Service NHS Foundation Trust. The vertical lines indicate the detection of the peaks by Public Health England for the following syndromes: influenza-like illness in week 1, 2017; acute respiratory illness in week 52, 2016.

Both peaks seen for the influenza season 2015/2016 and 2016/2017 correspond to the data collected by the ECDC. The comparison to the non-sentinel data shows an earlier peak of the ePCR data in the 2016/2017 flu season (Figure 3).

PHE recorded a peak of ILI consultations in week 1, 2017; ARI consultations peaked in week 52, 2016. This correlates temporally with the peaks seen in the daily summed data (week 1, 2017) and the weekly summed data (week 51, 2016), indicating that the seasonal influenza outbreak progresses similarly in both datasets and therefore allowing a direct comparison.

### The different mean-CR_d_ depending on window sizes

To establish the effect of different choices of d, the ascending area of pyrexia cases peak in 2016/2017 was used to calculate a sliding CR_d_ with varying d for the ascending slope where pyrexia cases increased.

**Figure 4:**
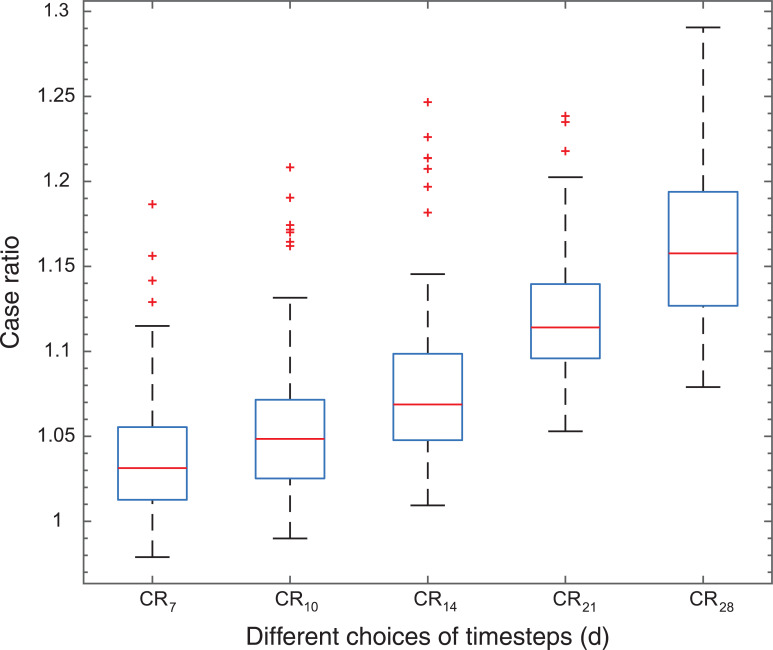
Figure 4. Comparison of different case ratios calculated with different time steps (d).

CR_21_ was chosen here as its values for the ascending slope were greater than 1, thus fitting the assumption that the slope represents an increase in case numbers. Although it included more outliers than CR_14_, it caused less delay than CR_28_, therefore it was chosen as a trade-off between timely detection and reduction of noise. To make the approach comparable between daily and weekly counts, the same window is used for both sampling rates, meaning a window of 21 days or 3 weeks for the daily and weekly counts, respectively (Figure 4).

### Daily detection

**Figure 5:**
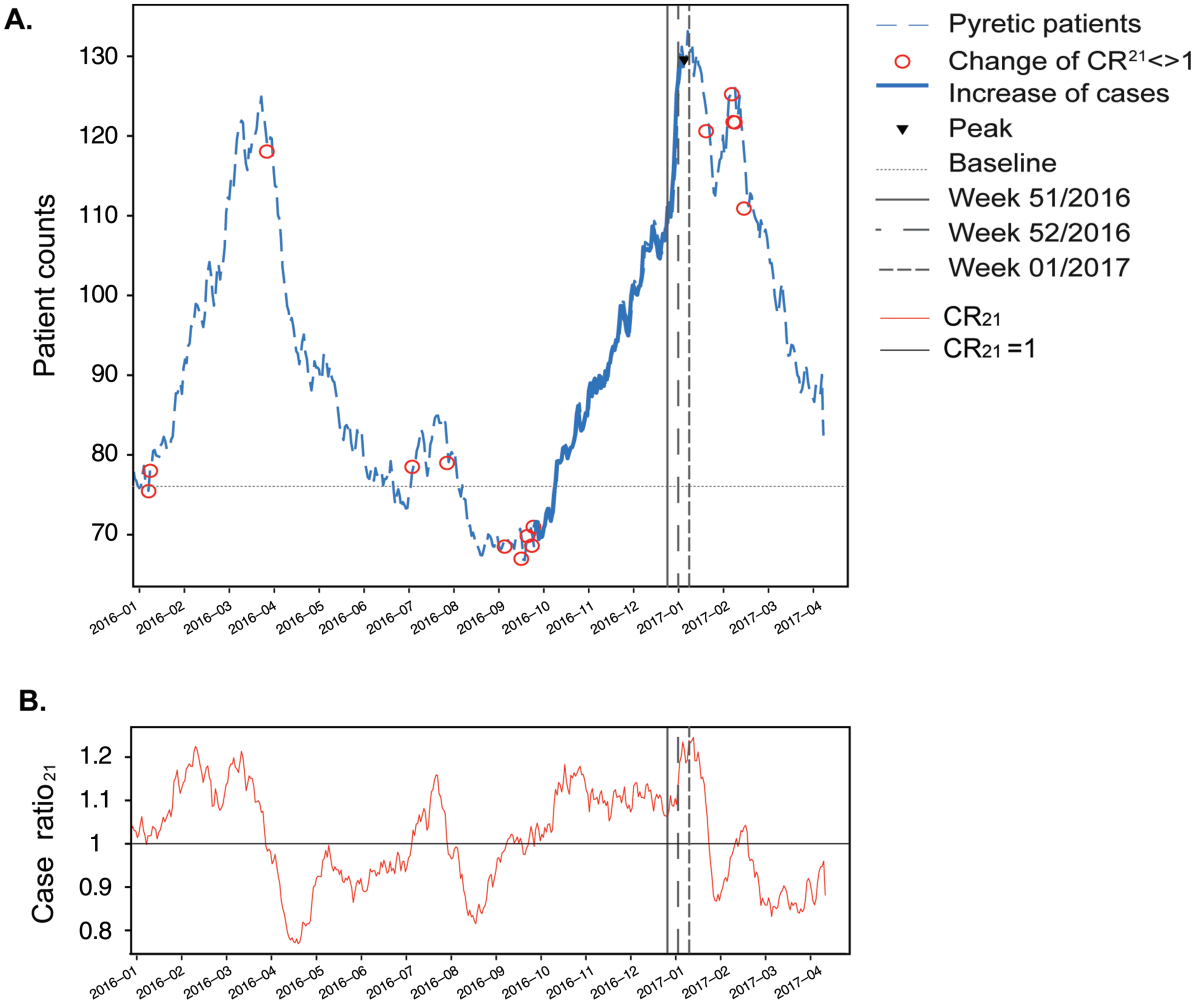
Figure 5. Daily pyrexia cases with CR_21_ indicated as circles (A) and CR_21_ values on the same timescale (B).

The smoothed daily pyretic case numbers peaked on 2 January 2017 (week 1, 2017). The peak was reached with 133 (18.5%) patients of 721 calls (fractions are caused by the smoothing process using the EMA). The start of the increase in pyrexia cases defined by a final change of the CR_21_ to > 1 was detected on 24 September 2016 (week 38, 2016) with 70 pyretic patients (11.2% of 633 calls, Figure 5). This value is 6.8% below the baseline (76.2) and within the standard deviation (23.5), which would not be detectable using a threshold method. This start of the seasonal increase of infections was detected earlier than influenza cases by the ECDC, which identified the start in week 46, 2016 (European Centre for Disease Prevention and Control, 2017).

### Weekly detection

**Figure 6:**
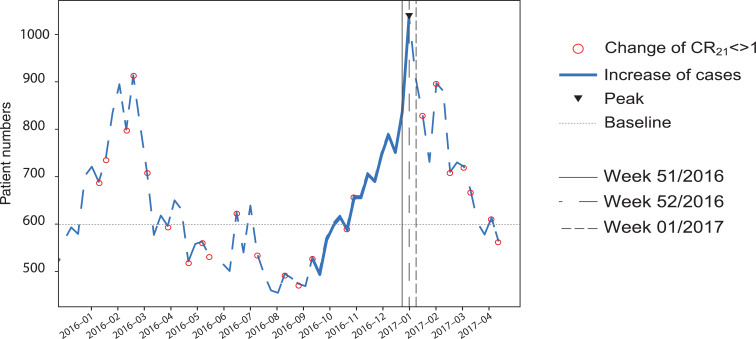
Figure 6. Weekly pyrexia cases with CR_21_ indicated as circles.

The weekly case numbers peaked in week 51, 2016 with 1042 (21.6%) pyretic patients of 4814 total calls. An increase of CR_21_ to > 1 was detected in week 39, 2016 with 570 (13.7%) cases of 4151 calls (Figure 6). The pyrexia count is 5.3% below the baseline (550) at the time of detection and within the standard deviation (134.56), hence would be difficult to detect with a threshold-based method.

As a comparison, the ECDC recorded the first cases in week 46, 2016 (European Centre for Disease Prevention and Control, 2017) corresponding to 693 (15.7%) cases of 4407 patients attended by ambulances. This pyrexia count at the date of detection by the ECDC is 15.5% above the baseline (550) but again within the standard deviation (134.6).

### Improving accuracy

**Figure 7:**
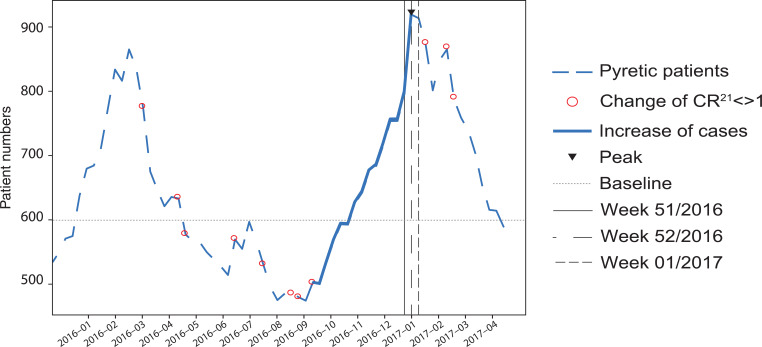
Figure 7. Weekly smoothed (3-week exponential weighted moving average) pyrexia cases, with CR_21_ indicated as circles.

The weekly data were smoothed using the EMA of 21 days (or three sample points) before the sliding CR_21_ was calculated (Figure 7). The smoothed weekly dataset peaked in week 51, 2016 with 919 (21.6%) pyretic patients of 4814 total calls. The final CR_21_ change to > 1 indicating the start of the outbreak was reached in week 44, 2017 with 485.5 (12.5%) pyretic patients of 3868 calls. This case number is 18.4% below the baseline (565) and within the standard deviation (122.3). Once again, the outbreak could not be detected using a threshold method as the number is below the baseline (565) as well as the mean (644.9).

## Discussion

Ambulance crews within SWASFT have collected data for 16% of the population in Devon and Cornwall within a year (Office for National Statistics, 2017). The collected data generally mirrored the gender characteristics of the population, but in terms of age, the SWASFT data had a higher proportion of elderly and infant patients. This reflects the fact that the elderly and the very young are more likely to require assistance by an ambulance. From these data, it was possible to establish that the pyrexia counts timely matched the seasonal influenza outbreak recorded by the ECDC.

It is reasonable to assume that a large number of pyrexia cases was due to seasonal influenza, as the peak of the pyrexia was simultaneous with the peak in ECDC-confirmed influenza cases. However, a proportion of cases will have been caused by other circulating infections. The temporal similarity of our data and the ECDC data shows that the use of temperature as the sole indicator of infection allows the unspecific monitoring of infectious diseases within the community.

The seasonal increase of fever cases was detected up to nine weeks before influenza cases were recorded by conventional methods employed by the ECDC. As the method described here does not specifically detect influenza, several contributing factors can be attributed to this finding. In the UK, the sentinel detection runs between October and March, thus it could not detect earlier cases. Furthermore, during winter many different infectious agents are circulating, including the common cold, all of which cause fever.

Due to its simplicity, the CR_d_ method is easy to deploy and will effectively detect a wide range of syndromes. By using case numbers of different symptoms, this method will also allow automatically distinguishing between different infections. For example, patients with a combination of pyrexia and diarrhoea could be monitored separately from patients experiencing pyrexia only. Therefore, as a continuation of this study, different symptoms will be combined to allow the monitoring of several syndromes in parallel.

The necessity to choose a time frame raises similar questions as threshold-based methods, on how to decide on the best time frame. Therefore, this flexibility makes the CR_d_ method adaptable to the data and transmission rates allowing reduction of false positives in noisy data.

In practice, this method could be further developed to allow the surveillance of several different syndromes but can also be used to anticipate spikes in ambulance call rates as well as hospital admissions. It might also lead the way to predict the length and magnitude of a detected outbreak.

### Limitations

The collected data could have included patients with multiple ambulance attendances a year, which cannot be accounted for, as no patient identifying data were extracted.

An unknown proportion of pyrexia cases will be caused by other infections, although it can be expected that a large fraction was caused by the circulating seasonal influenza virus. Furthermore, the comparison data originated from different geographic populations (Devon and Cornwall vs. England) and were compared to confirmed influenza diagnoses.

Although the proposed method does not rely on a threshold to detect an increase in cases, it still requires the user to define the value of d, which will normally require some knowledge about the transmission rate of the monitored infection.

## Conclusion

Data from ambulance service ePCRs correlate with the sentinel data collected by the ECDC, allowing these data to inform an EED system. The detection of events occurred earlier compared to the ECDC, but does not distinguish between infectious agents. The move to digital patient records makes it possible to monitor the large proportions of the population at high sample rates, and for several syndromes simultaneously, making it an ideal data source for an EED system.

## Acknowledgements

Thanks go to the South Western Ambulance Service NHS Foundation Trust’s research and development team and especially Kim Kirby who assisted in gaining access to the data.

## Author contributions

TR: Concept and study design, data analysis and interpretation, drafting of the work, approval of final work.

MB: Data interpretation and analysis improvements, critical revision of the work, approval of final work.

## Conflict of interest

None declared.

## Ethics

Not required.

## Funding

None.
